# Two new species of the genera *Mysmena* and *Trogloneta* (Mysmenidae, Araneae) from Southwestern China

**DOI:** 10.3897/zookeys.303.4808

**Published:** 2013-05-21

**Authors:** Yucheng Lin, Shuqiang Li

**Affiliations:** 1 Key Laboratory of Bio-resources and Eco-environment (Ministry of Education), College of Life Sciences, Sichuan University, Chengdu, Sichuan 610064, China; 2 Institute of Zoology, Chinese Academy of Sciences, Beijing 100101, China

**Keywords:** Taxonomy, diagnosis, description, forest, etymology

## Abstract

Two new spider species of the family Mysmenidae Petrunkevitch, 1928 are reported from Southwestern China, i.e., *Mysmena wawuensis* **sp. n.** (male and female) from Sichuan and *Trogloneta yuensis* **sp. n.** (male) from Chongqing. Diagnoses and illustrations of the new species are provided.

## Introduction

Mysmenidae is a small family of minute araneoid spiders. Although the family Mysmenidae is distributed worldwide, it is one of the least-studied family-level groups among orb-weaving spiders, and its diversity is grossly undersampled due to their small size (0.7–3 mm) and cryptic life style (Lopardo et al. 2011). Mysmenids mainly occur in leaf litter and other cryptic places in very humid habitats (Lopardo & Coddington 2005), and even in caves. Their distribution ranges throughout the tropical or subtropical regions of Eurasia, America and Africa. According to the latest records, a total of 123 species and 23 genera were reported in the family Mysmenidae (Platnick 2013). Up to present, 30 species placed in 9 genera have been described in China (Yin et al. 2004; Ono 2007; Lin and Li 2008; Miller et al. 2009).

The genus *Mysmena* was erected by Simon in 1894 initially as a genus of the family Theridiidae with the type species *Theridion leycoplagiatum* Simon, 1879; later transferred to the Symphytognathidae by Forster (1959), and then to the family Mysmenidae by Forster and Platnick (1977). To date, 23 *Mysmena* species have been reported worldwide (Platnick 2013), including 12 species from China which is about a half of all species of the genus *Mysmena* (Ono 2007; Lin and Li 2008; Miller et al. 2009).

The genus *Trogloneta* was established and placed in the family Theridiidae by Simon in 1922 for a minute spider from caves in France, *Trogloneta granulum* (“*Troglonata”* was misspelled in the original description, see Simon 1926: 313) (Brescovit & Lopardo 2008). Gertsch (1960) transferred this genus to the family Symphytognathidae, and then Forster & Platnick (1977) put it in the family Mysmenidae. Until now there is no consistent diagnosis for *Trogloneta*, Brescovit and Lopardo (2008) proposed that this genus can be distinguished from other mysmenids by the following combination of features: AME smaller than ALE; one femoral spot on leg I on both males and females; one male clasping spine on metatarsus I; males with highly elevated and conical carapace, and male pedipalp very large. Additional diagnostic characters may include the clustering of eyes around the apex of the carapace in males (Fig. 8A–B; Lin & Li 2008: figs 16A–B, 19A–B) and the abdomen usually pointed dorsal-posteriorly (the exception is *Trogloneta denticocleari* Lin & Li, 2008, which has a globose abdomen).

At present, 9 *Trogloneta* species are known from America, Europe, Asia and some Atlantic islands (Platnick 2013), including two species reported from China (Lin & Li 2008): one found in caves from the Yunnan-Guizhou Plateau, another found at the canopy of Xishuangbanna tropical rainforest.

In this paper we described two new species of genera *Mysmena* and *Trogloneta* from Wawu Mt., Sichuan and Jinyun Mt., Chongqing of Southwestern China, *Mysmena wawuensis* sp. n. and *Trogloneta yuensis* sp. n.

## Material and methods

Specimens were examined and measured under an Olympus SZX7 stereomicroscope. Further details were studied under an Olympus BX43 compound microscope. All drawings were made using a drawing tube attached to Olympus BX43 compound microscope, and then inked on ink jet plotter paper. Photos were taken with a Canon EOS 60D wide zoom digital camera (8.5 megapixels). The images were montaged using Helicon Focus 3.10 software. Male pedipalpi and female genitalia were examined and illustrated after they were dissected and detached from the spiders’ bodies. Vulvae were removed and treated in lactic acid before illustration. To reveal the course of spermatic duct, the pedipalpal bulb was also treated in lactic acid and mounted in Hoyer’s Solution. Left pedipalp of male spiders was illustrated. All specimens are preserved in 85% ethanol solution.

All measurements are in millimeters. Leg measurements are given as: total length (femur, patella, tibia, metatarsus, and tarsus). The terminology mostly follows Lopardo et al. (2011). The abbreviations used in text including: AER – anterior eye row; ALE – anterior lateral eye; AME – anterior median eye; PER – posterior eye row; PLE – posterior lateral eye; PME – posterior median eye. All specimens are deposited in the Zoological Department of the School of Life Science, Sichuan University Museum (SCUM) in Chengdu.

## Taxonomy

### *Mysmena* Simon, 1894

**Type species.** *Theridion leycoplagiatum* Simon, 1879

#### Mysmena wawuensis sp. n.

urn:lsid:zoobank.org:act:FF5B96D7-39D7-4F3E-816D-521A57F1413C

http://species-id.net/wiki/Mysmena_wawuensis

Figs 1 –7 , 13

##### Material examined.

Holotype: CHINA, Sichuan: Hongya County, Wawu Mt. National Forest Park, Gufuping, 29°40.114'N, 102°57.515'E, elevation ca 1929 m, 27 June 2012, by hand collection, Yucheng Lin leg., male (SCUM).

Paratypes: [same data as holotype] (SCUM), 2 females.

##### Etymology.

The specific name is taken from the type locality; adjective.

##### Diagnosis.

This new species is similar to *Mysmena goudao* Miller, Griswold & Yin, 2009 (see Miller et al. 2009: 39, figs 21F–G, 27A–E, 28A–B, 29A–F) in male pedipalpal shape and female genital configuration. Male differs from the latter by the presence of a subdistal cymbial process (Figs 3D–E, 5A, 6E), a subdistal-ventral marcoseta on the pedipalpal femur (Figs 2A–B, 5A–B), the absence of cymbial groove (Figs 3D–E, 6D–E). Female by a small, weakly sclerotized scape (Figs 4B–C, 7B–C), a paired rugose accessory bursae (Figs 4C, 7C) and twisted course of spermathecae (Figs 4C, 7C).

##### Description.

**Figure 1.**
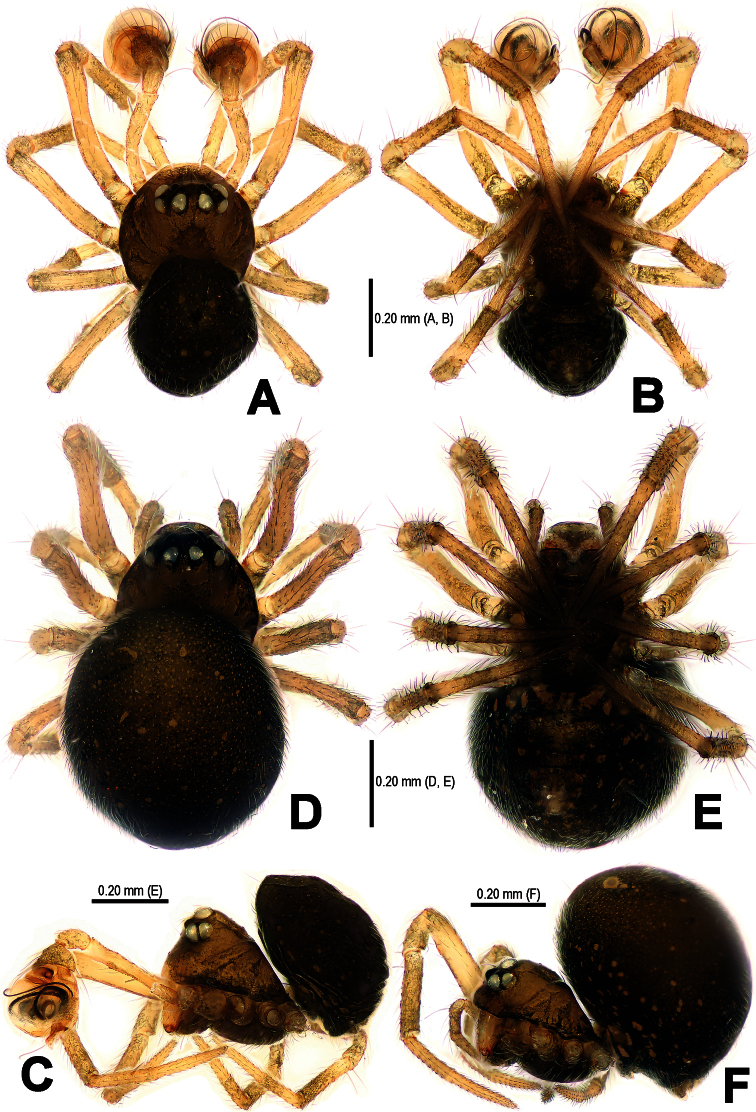
*Mysmena wawuensis* sp. n., male holotype (**A–C**) and female paratype (**D–F**). **A–F** Habitus. **A, D** dorsal view **B, E** ventral view **C, F** lateral view.

**Male** (holotype). Somatic characters see Fig. 1A–C. Coloration: Prosoma brown centrally, dark marginally. Sternum black. Opisthosoma black, with tiny yellow speckles.

Measurement: Total length 0.60. Prosoma 0.36 long, 0.35 wide, 0.32 high. Opisthosoma 0.36 long, 0.32 wide, 0.39 high. Clypeus 0.12 high. Sternum 0.25 long, 0.21 wide. Length of legs [total length (femur + patella + tibia + metatarsus + tarsus)]: I 1.14 (0.36, 0.14, 0.25, 0.18, 0.21); II 0.97 (0.30, 0.13, 0.21, 0.14, 0.19); III 0.76 (0.21, 0.11, 0.13, 0.13, 0.18); IV 0.93 (0.29, 0.13, 0.20, 0.14, 0.17).

Prosoma (Fig. 1A, C): Carapace near round. Cephalic pars elevated, sharply vertical forward and slope backward. Ocular area at apex, dark. Eight eyes in two rows. AME black, others white. ALE and PLE contiguous. AME smallest, ALE largest. ARE slightly procurved, PRE straight. Chelicerae yellow, small, as long as endites (Fig. 1C).

Legs: Femora pale yellow, other segments yellow proximally, gray distally. Leg formula: I-II-IV-III. Leg I with a distal metatarsal clasping macroseta prolaterally on 1/3 position. Leg I and II with a subdistal sclerotized femoral spot ventrally. Patellae I–IV with a dorsal seta distally. Tibiae I–IV with a dorsal seta proximally, and with 3 trichobothria. Metatarsi I–IV with only one trichobothrium.

Opisthosoma (Fig. 1A–C): Globular dorsally. Spinnerets dark, the anteriors larger than the posteriors. Colulus indistinct. Anal tubercle grey.

**Figure 2.**
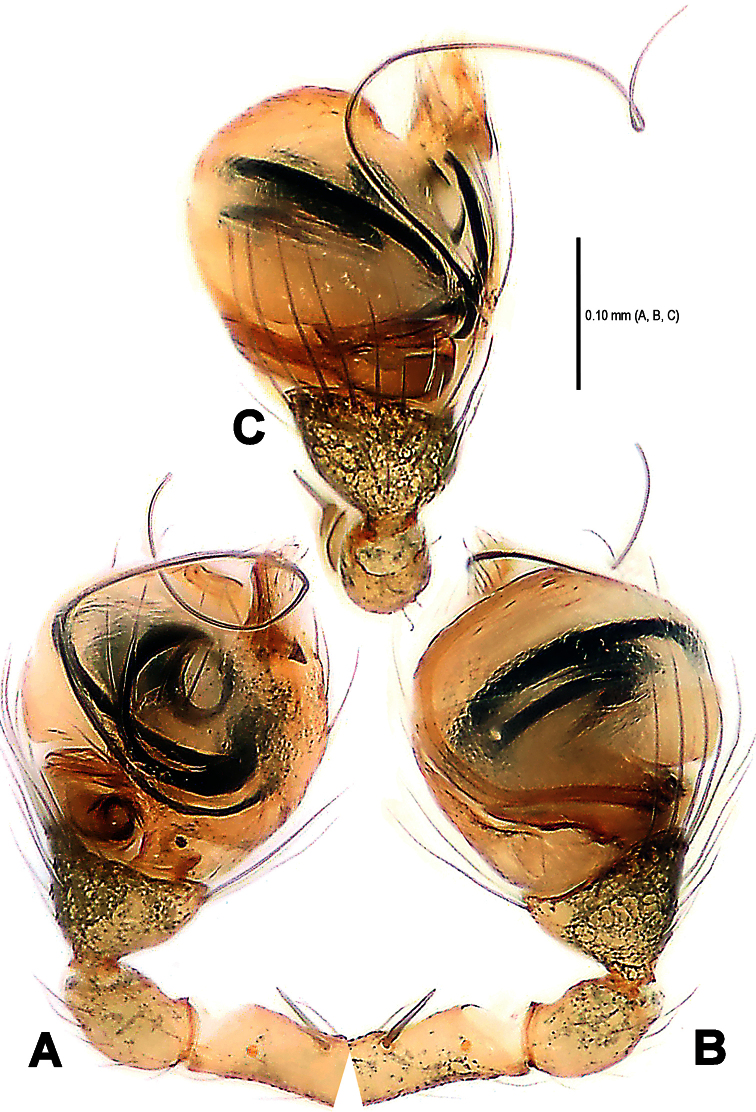
*Mysmena wawuensis* sp. n., male holotype. **A–C** Left pedipalp. **A** prolateral view **B** retrolateral view **C** dorsal view.

**Figure 3.**
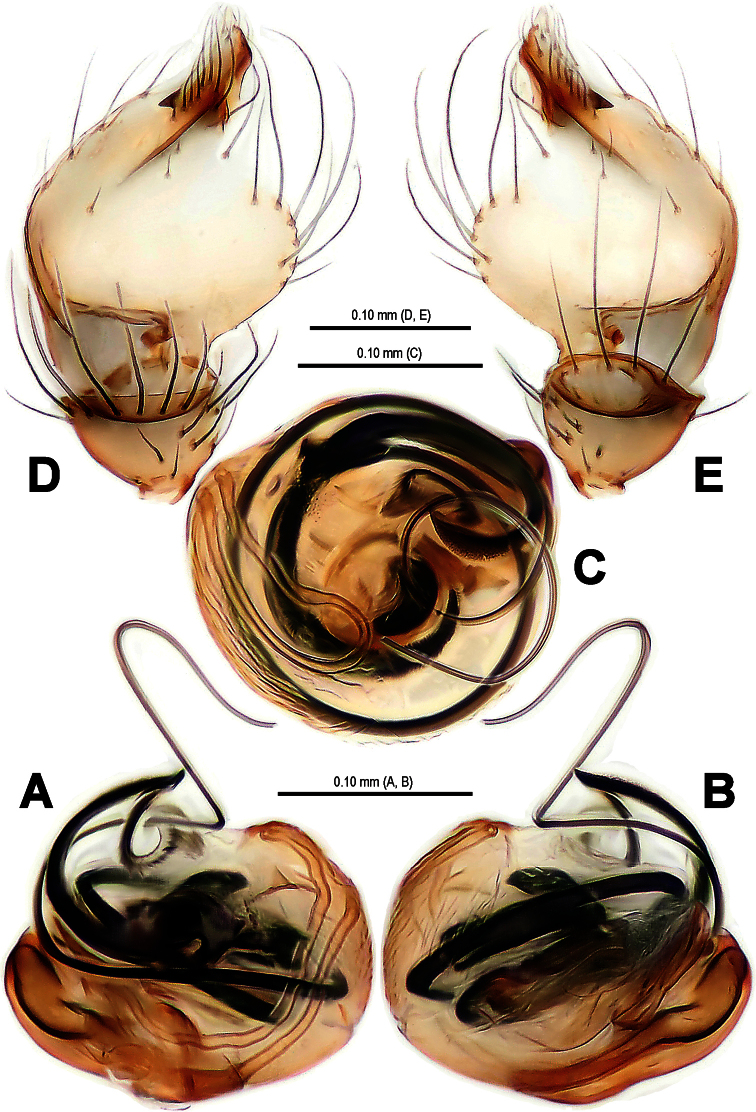
*Mysmma wawuensis* sp. n., male holotype. **A–C** Pedipalpal bulb **D–E** Cymbium. **A** ventral view **B** dorsal view **C** apical view **D** ventral view **E** dorsal view.

Pedipalp (Figs 2–3, 5–6): Femur long, with a subdistal macroseta ventrally (Figs 2A–B, 5A–B). Patella short, with a few setae. Tibia swollen, bowl-shaped, covered with long setae on distal margin ventrally and dorsally (Figs 3D–E, 6D–E). Cymbium membranous, wide, arisen from tibial margin ventrally (Fig. 6E), paracymbium attached with long setae along prolateral margin, a sclerotized cymbial process subdistally, a row of setae on cymbial fold subdistally and a primary cymbial conductor distally (Figs 3D–E, 6D–E). Tegulum rugose, translucent (Figs 2C, 3A–C). Spermatic duct visible through subtegulum (Figs 3A–C, 6A–C). Embolus long, thin and sparal (Figs 3C, 6C), coiling into four loops. Embolic end exceeded apex of cymbium (Figs 2C, 5A–C).

**Female** (one of paratypes). Somatic characters see Fig. 1D–F. Coloration: Same as in male.

Measurement: Total length 0.75. Prosoma 0.36 long, 0.32 wide, 0.30 high. Opisthosoma as in male, 0.54 long, 0.50 wide, 0.61 high. Clypeus 0.05 high, distinctly lower than in male. Sternum 0.23 long, 0.21 wide. Length of legs [total length (femur + patella + tibia + metatarsus + tarsus)]: I 1.05 (0.34, 0.14, 0.21, 0.16, 0.20); II 0.93 (0.29, 0.13, 0.18, 0.14, 0.19); III 0.77 (0.23, 0.11, 0.13, 0.13, 0.17); IV 0.99 (0.30, 0.13, 0.20, 0.16, 0.20).

Prosoma (Fig. 1D, F): Carapace near pear-shaped. Cephalic part lower than in male. Eyes arrangement, chelicerae and endites as in male.

Legs: Color, number of trichobothria same as in male, except for leg I without distal metatarsal clasping macroseta prolaterally. Sclerotized femoral spot present at leg I and II as in male. Leg formula: I-IV-II-III.

Opisthosoma (Fig. 1D–F): Globose dorsally. Spinnerets grey, the anteriors larger than the posteriors. Colulus small, pale.

**Figure 4.**
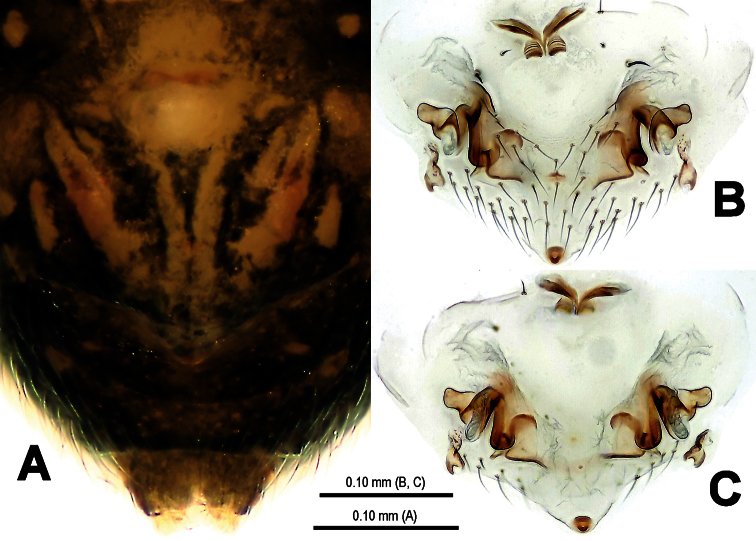
*Mysmena wawuensis* sp. n., female paratype. **A** Epigynum, ventral view **B** Epigynum (lactic acid-treated), ventral view **C** Vulva (cleared), dorsal view.

Epigynum (Figs 4, 7): Large, weakly sclerotized, darkish. Epigynal area covered with short setae (Fig. 4B). A small, sclerotized scape stands on epigynal posteromargin mesially (Fig. 4B–C). Spermathecae short clubbed, weakly sclerotized, twisted, attached with membranous, rugose accessory bursae (Figs 4C, 7C). Fertilization ducts short, connected with spermathecae and accessory bursa. Copulatory ducts long, curved, weekly sclerotized, derives from inner side of spermathecae ventrally (Figs 4C, 7C).

##### Distribution.

Known only from the type locality (Fig. 13).

**Figure 5.**
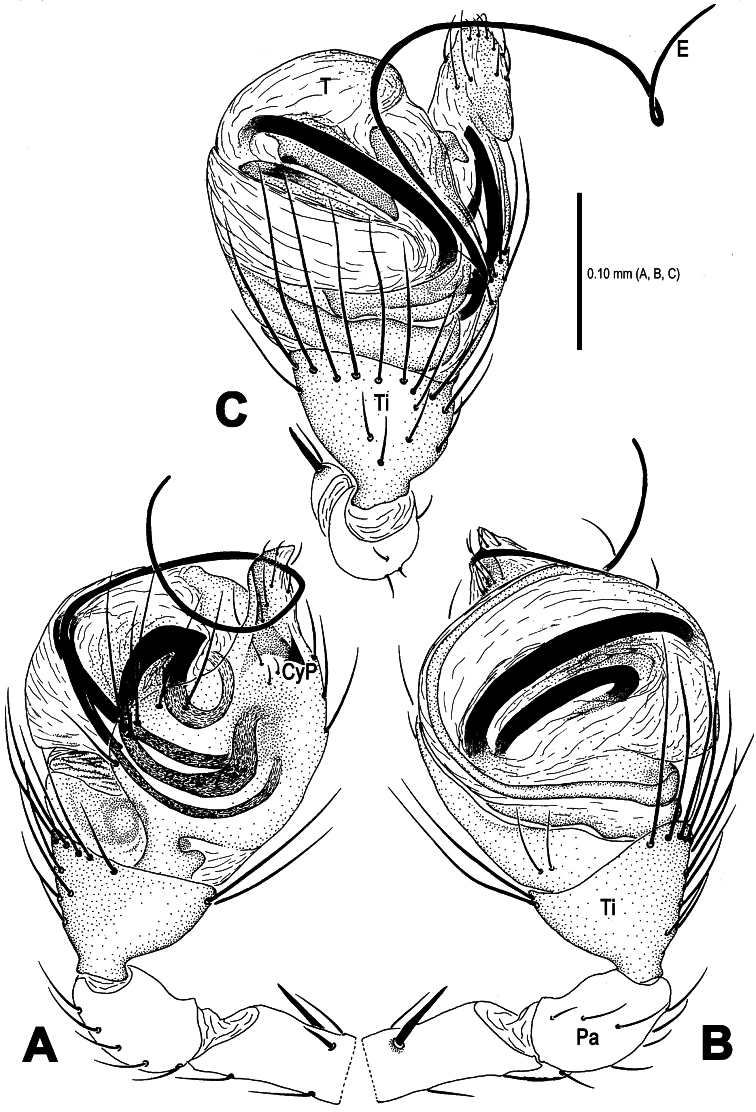
*Mysmena wawuensis* sp. n., male holotype. **A–C** Left pedipalp. **A** prolateral view **B** retrolateral view **C** dorsal view. Abbrs.: **CyP** cymbial process; **E** embolus; **Pa** patella; **T** tegulum; **Ti** tibia.

**Figure 6.**
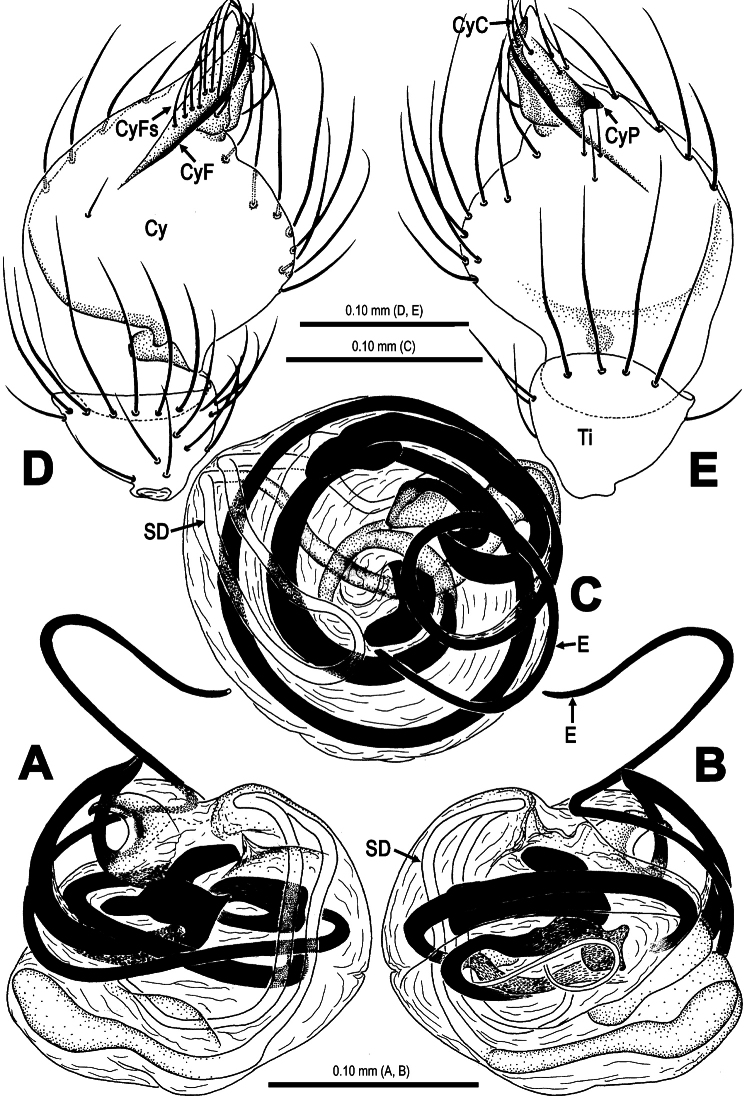
*Mysmena wawuensis* sp. n., male holotype. **A–C** Pedipalpal bulb, **D–E** Cymbium. **A** ventral view **B** dorsal view **C** apical view **D** ventral view **E** dorsal view. Abbrs.: **Cy** cymbium; **CyC** cymbial conductor; **CyF** cymbial fold; **CyFs** setae on cymbial fold; **CyP** cymbial process; **E** embolus; **SD** spermatic duct; **Ti** tibia.

**Figure 7.**
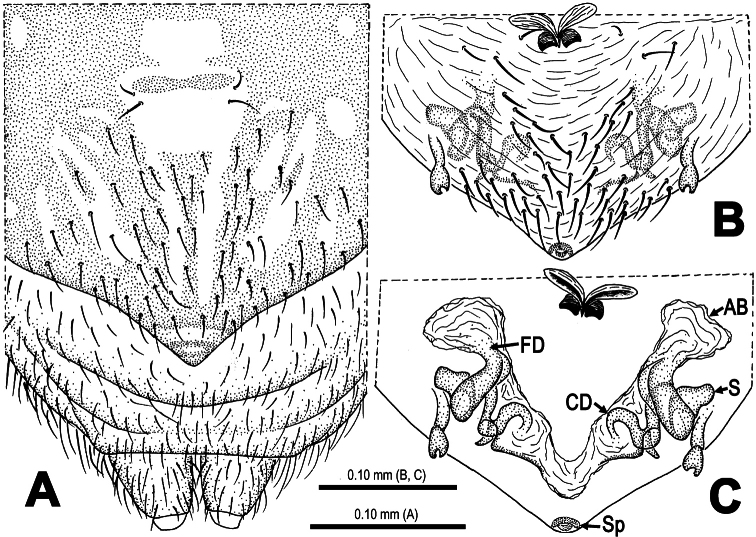
*Mysmena wawuensis* sp. n., female paratype. **A** Epigynum, ventral view **B** Epigynum (lactic acid-treated), ventral view **C** Vulva (cleared), dorsal view. Abbrs.: **AB** accessory bursa; **CD** copulatory duct; **FD** fertilization duct; **S** spermatheca; **Sp** scape.

### *Trogloneta* Simon, 1922

**Type species.** *Trogloneta granulum* Simon, 1922

#### Trogloneta yuensis sp. n.

urn:lsid:zoobank.org:act:47B062D1-CCC8-4C2B-978B-6ABF9B135CDF

http://species-id.net/wiki/Trogloneta_yuensis

Figs 8 –13

##### Material examined.

Holotype: CHINA, Chongqing: Beibei District, Jinyun Mt., Guankou, 29°50.261'N, 106°23.811'E, elevation ca 531 m, 5 April 2010, by sieving, Zhisheng Zhang leg., male (SCUM).

##### Etymology.

The specific name is taken from the type locality; adjective. Yu is short name for Chongqing.

##### Diagnosis.

**Figure 8.**
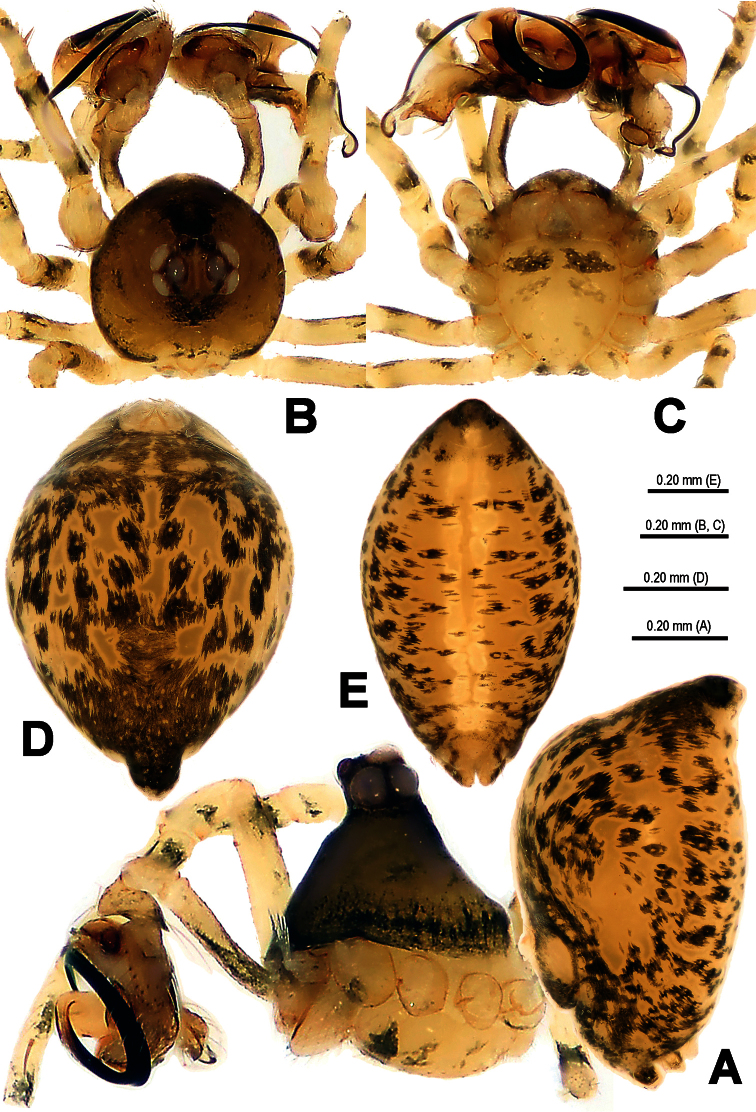
*Trogloneta yuensis* sp. n., male holotype. **A** Habitus, lateral view **B** Prosoma, dorsal view **C** Ditto,ventral view **D** Opisthosoma, dorsal view **E** Ditto, posterior view.

**Figure 9.**
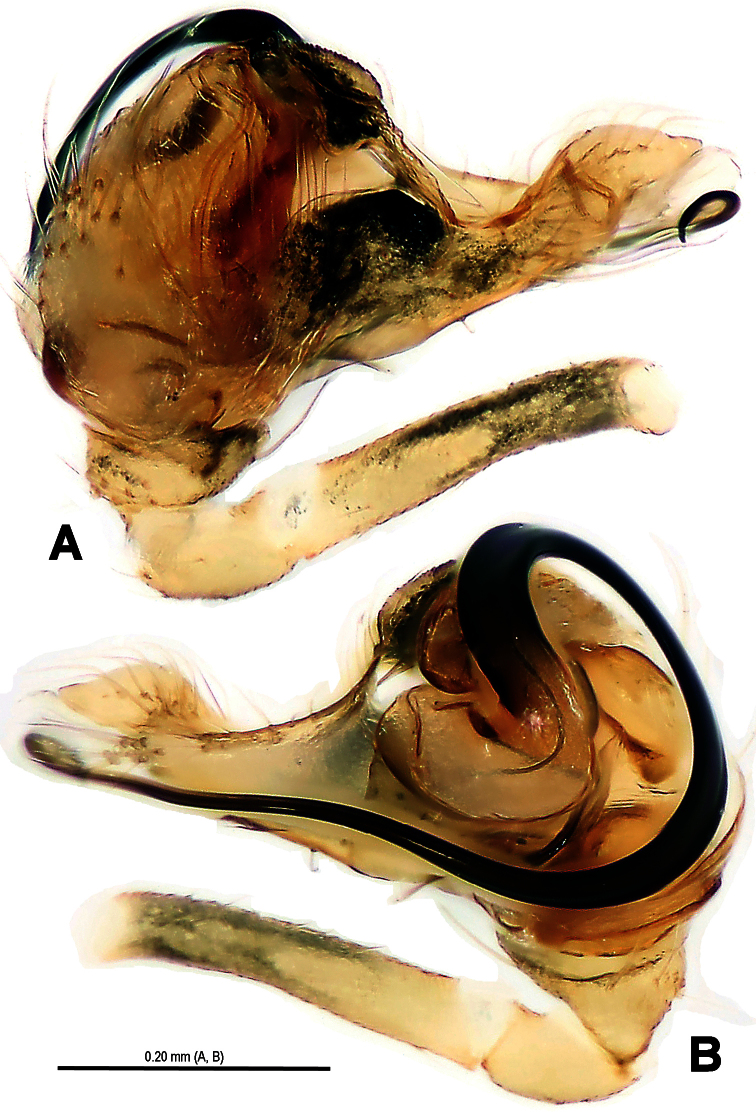
*Trogloneta yuensis* sp. n., male holotype. **A** Left pedipalp, retrolateral view **B** Ditto, prolateral view.

**Figure 10.**
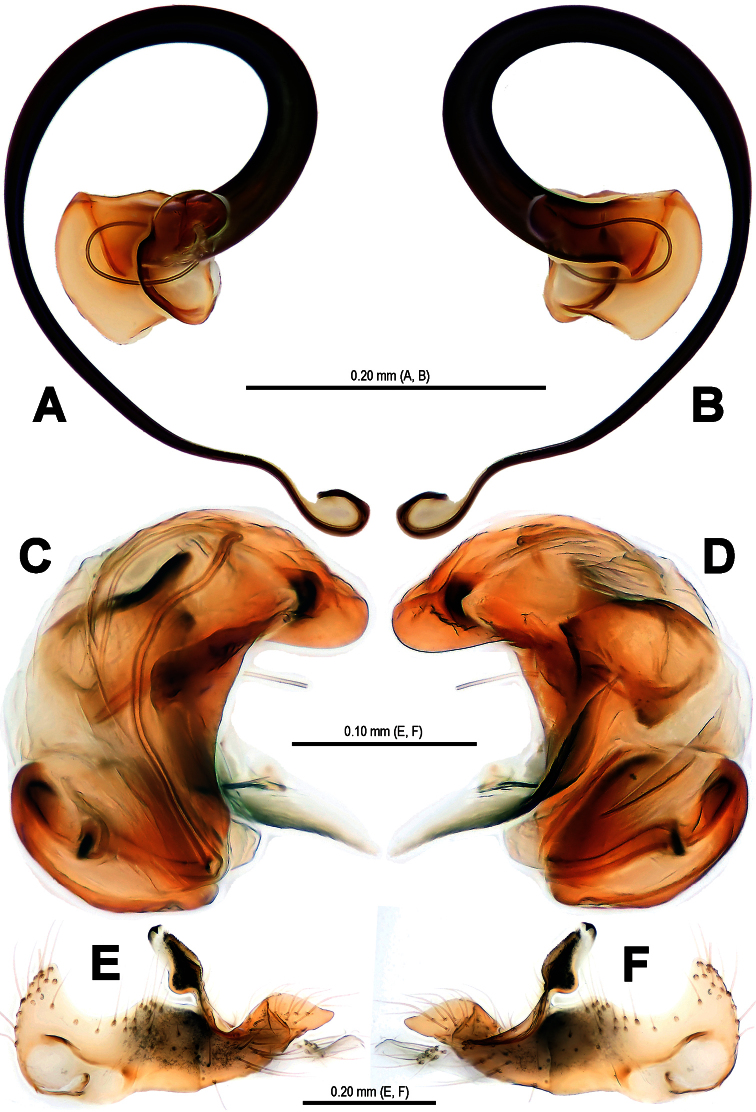
*Trogloneta yuensis* sp. n., male holotype. **A** Embolus, ventral view **B** Ditto, dorsal view **C** Pedipalpal bulb (excluding embolus), ventral view **D** Ditto, dorsal view **E** Cymbium, dorsal view **F** Ditto, ventral view.

**Figure 11.**
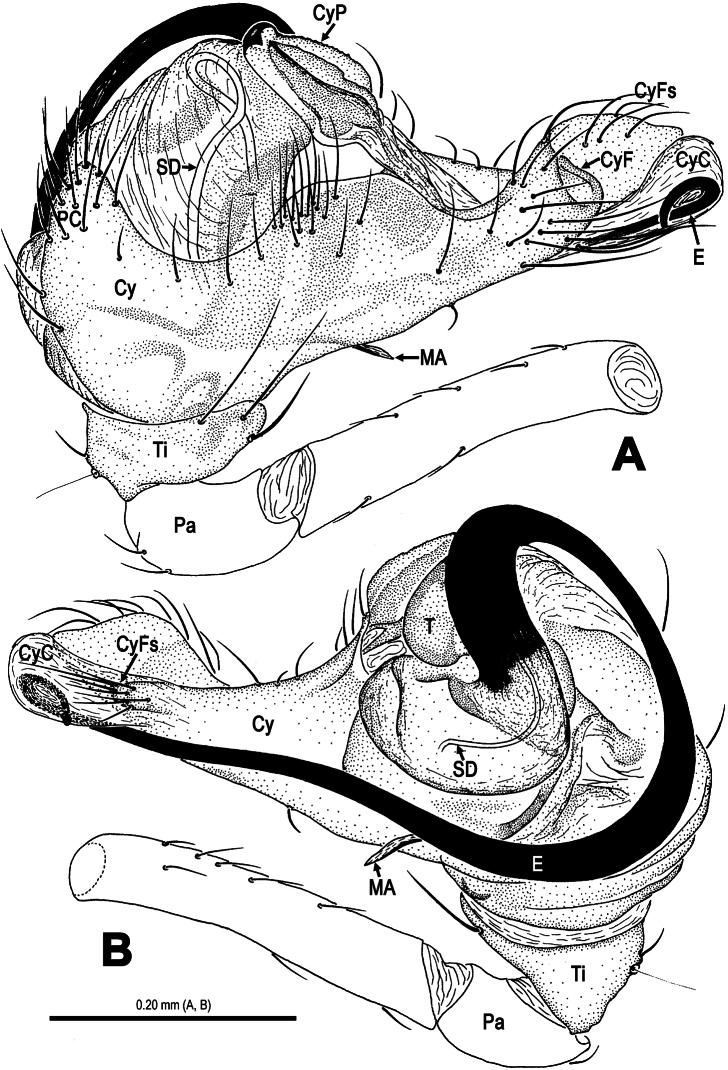
*Trogloneta yuensis* sp. n., male holotype. **A** Left pedipalp, retrolateral view **B** Ditto, prolateral view. Abbrs.: **Cy** cymbium; **CyC** cymbial conductor; **CyF** cymbial fold; **CyFs** setae on cymbial fold; **CyP** cymbial process; **E** embolus; **MA** median apophysis; **Pa** patella; **PC** paracymbium; **SD** spermatic duct; **T** tegulum; **Ti** tibia.

**Figure 12.**
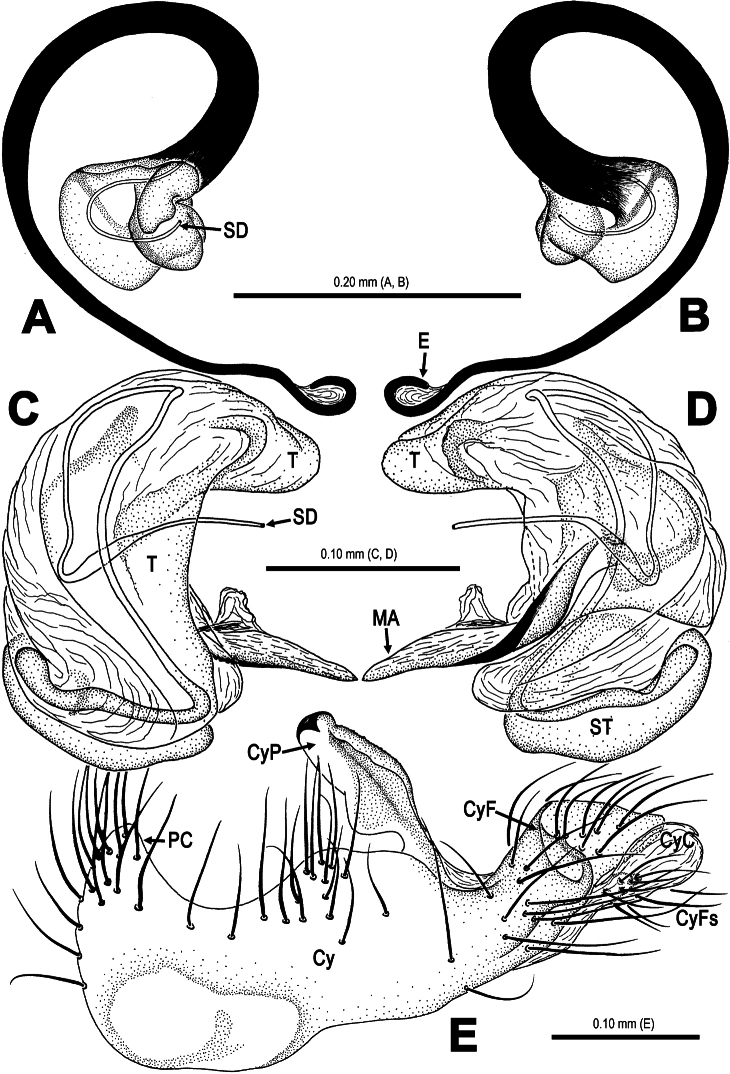
*Trogloneta yuensis* sp. n., male holotype. **A–B** Embolus. **A** ventral view **B** dorsal view **C–D** Pedipalpal bulb (excluding embolus) **C** ventral view **D** dorsal view **E** Cymbium, dorsal view. Abbrs.: **Cy** cymbium; **CyC** cymbial conductor; **CyF** cymbial fold; **CyFs** setae on cymbial fold; **CyP** cymbial process; **E** embolus; **MA** median apophysis; **Pa** patella; **PC** paracymbium; **SD** spermatic duct; **ST** subtegulum; **T** tegulum.

This new species has the following combinations of typical generic features: AME dark, smaller ALE (Fig. 8B); eyes at the apex (Fig. 8A); male leg I with a femoral spot and a metatarsal clasping spine; highly elevated and conical carapace (Fig. 8A); male pedipalp large (Fig. 8B–C). All indicating that this species belongs to the genus *Trogloneta*. This new species is similar to *Trogloneta denticocleari* Lin & Li, 2008 (see Lin and Li 2008: 513, figs 16A–E, 17A–C) in habitus (Fig. 8A), eyes arrangement (Fig. 8B), pedipalp shape (Figs 9A–B, 11A–B), cymbial configuration (Figs 11A, 12E) and a trichobothrium present at pedipalpal tibia (Fig. 11A–B), but distinguished from the latter by a long, distally hooked embolus attaching accessory membrane (Figs 10A–B, 12A–B), a long fingerlike median apophysis (Figs 10C–D, 12C–D), a laminar cymbial conductor (Fig. 12E), a distally aquiline, basally constricted cymbial process (Figs 10E–F, 11A, 12E) and a dorsal-posterior opisthosomal tubercle (Fig. 8A, D–E).

##### Description.

**Male** (holotype). Somatic characters see Fig. 8A–E. Coloration: Prosoma yellow centrally, dark marginally. Clypeus black. Sternum yellow, with a pair of shoulder dark speckles. Opisthosoma yellow, with irregular dark spots.

Measurement: Total length 1.01. Prosoma 0.45 long, 0.45 wide, 0.59 high. Opisthosoma 0.54 long, 0.55 wide, 0.95 high. Clypeus 0.32 high. Sternum 0.31 long, 0.29 wide. Length of legs [total length (femur + patella + tibia + metatarsus + tarsus)]: I 1.42 (0.43, 0.17, 0.32, 0.29, 0.21); II 1.15 (0.38, 0.16, 0.23, 0.22, 0.16); III 0.96 (0.29, 0.13, 0.20, 0.18, 0.16); IV 1.15 (0.36, 0.14, 0.26, 0.22, 0.17).

Prosoma (Fig. 8A–C): Carapace near round. Cephalic pars sharply elevated, slope forward and backward. Ocular area at apex. Eight eyes in two rows. AME black, others white. AME smallest, ALE largest. ALE>PLE>PME>AME. ALE, PME and PLE contiguous. ARE procurved, PRE strongly procurved. Chelicerae pale, small, shorter than endites (Fig. 8A), fang furrow with 2 promaiginal and 1 retromarginal teeth.

Legs: Femora and other segments pale yellow mesially, but grey proximally and distally. Leg formula: I-II-IV-III. Leg I with a subdistal sclerotized femoral spot ventrally and a submesial metatarsal clasping macroseta prolaterally. Patellae I–IV with a dorsal seta distally. Tibiae I–IV with a dorsal seta proximally. Tibiae I, II and IV with 3 trichobothria, but 4 on tibia III. Metatarsi I–IV lack trichobothrium.

Opisthosoma (Fig. 8A, D–E): elliptic dorsally, fusiform posteriorly, triangular laterally, with a tubercle at rear. Spinnerets grey, the anteriors larger than the posteriors. Colulus small, tongue-shaped. Anal tubercle pale.

Pedipalp (Figs 9–12): Large, strongly sclerotized. Femur as 2.5 times long as patella (Fig. 9A, B). Patella short, with a few setae. Tibia wider than long, nearly cup-shaped, covered with a dorsal trichobothrium and a few marginal long setae ventrally (Figs 11A–B). Cymbium large (Figs 10E–F, 12E), membranous, paracymbium flattened, covered with dense long setae. A long cymbial process (aquiline distally, constricted proximally) arisen from inner side subdistal margin (Fig. 12E). Cymbial fold distinctly, with long setae. Distal primary cymbial conductor membranous, translucent, attaching with a cluster of setae (Fig. 12E). Tegulum smooth, sclerotized (Fig. 10C–D). Spermatic duct long, visible through subtegulum (Fig. 11C–D). A long, fingerlike median apophysis starts at the junction between tegulum and subtegulum (Figs 10D, 11D). Embolus long, arched, strongly sclerotized, gradually diminishing from base to end (Figs 9B, 12A–B). Embolic end unciform, with accessory membrane (Fig. 12A–B), hidden behind cymbial conductor (Figs 9B, 11B).

**Female**. Unknown.

##### Distribution.

**Figure 13.**
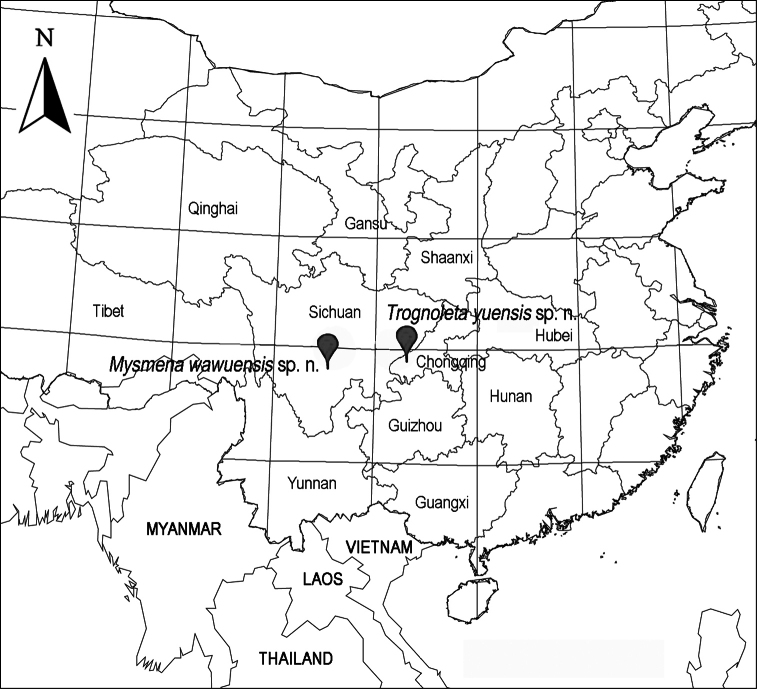
Distributional records of two new mysmenid species from China.

Known only from the type locality (Fig. 13).

## Supplementary Material

XML Treatment for Mysmena wawuensis

XML Treatment for Trogloneta yuensis
